# A Community-Partnered Cancer Disparities Research Collaborative Model for Engaging Members of Low-Income Black Communities in Cancer Research

**DOI:** 10.1007/s10900-025-01494-2

**Published:** 2025-06-29

**Authors:** Carolyn M. Tucker, Kirsten G. Klein, Guillermo Wippold, Lakeshia Cousin, Staja Q. Booker, Ji-Hyun Lee, Juanita Miles Hamilton, Kevin Arenus, Danielle E. Jake-Schoffman, Kevin A. Thorpe, Adrian S. Taylor, Ron Rawls, Kenyarda Feathers, Jaclyn Hall

**Affiliations:** 1 University of Florida, Gainesville, FL USA; 2 University of South Carolina, Columbia, SC USA; 3 Faith Missionary Baptist Church, Gainesville, FL USA; 4 Springhill Missionary Baptist Church, Gainesville, FL USA; 5 Greater Bethel AME Church, Gainesville, FL USA; 6 Williams Temple Church of God in Christ, Gainesville, FL USA

**Keywords:** Black adults, Cancer, Community based participatory research, Health equity, Implementation

## Abstract

Black adults experience the highest incidence and/or mortality rates for many commonly diagnosed cancers; yet they are underrepresented in cancer basic, applied, and translational research. This underrepresentation is greatest among Black adults living in low-income communities. There are many barriers to these individuals participating in cancer research, including limited effective research engagement strategies. This paper describes the University of Florida (UF) Health Cancer Center Community-Partnered Cancer Disparities Research Collaborative (CDRC) model and its implementation. The UF CDRC model consists of human and physical infrastructure for the sustained engagement of Black adults within low-income communities in cancer research. The CDRC model is culturally sensitive and informed by the community-based participatory research approach, which requires equitable partnerships between scientific researchers and community members. The human infrastructure of the CDRC model includes faculty and community researchers, pastors, and culturally diverse university students. The physical infrastructure of the CDRC model consists of 10 predominantly Black churches and a community-based multipurpose building, all of which are community outreach and research sites. Examples of evidence supporting the use of the CDRC model includes (a) the successful recruitment, training, and retention of trusted community members and leaders as major research partners in the CDRC, and (b) the pipeline development of culturally sensitive student and faculty health equity researchers. The CDRC model is a novel, replicable infrastructure model for the sustained engagement of Black adults within low-income communities in cancer prevention, treatment, and survivorship research. It is dedicated to reducing cancer disparities that plague Black communities.

## Background

Common cancers, such as breast, prostate, lung and colorectal cancer are disproportionately more prevalent among Black adults [1] and contribute to a high rate of premature mortality [1], compared to other racial/ethnic groups in the United States. Despite this high premature mortality rate, Black adults are underrepresented in clinical trials and other health research that aim to prevent cancer, develop effective treatments for cancer, and/or improve quality of life among cancer survivors. Specifically, clinical trial enrollments remain at 4–6% for Black adults, with even lower rates for Black men [2, 3].

The National Cancer Institute (NCI) and the American Cancer Society have called attention to the urgent need for research aimed at preventing cancer and reducing cancer disparities [4, 5]. Barriers to such research include limited effective strategies for recruiting and engaging Black adults to be research participants and a paucity of cancer education and screening interventions that are tailored to facilitate enrollment for Black participants [6–8]. These barriers are particularly salient among Black adults living in low-income communities because of the social determinants health (SDoH) that have a disproportionately negative impact on all aspects of their lives, including participation in cancer and cancer-related health research [9, 10].

The SDoHs that must be considered in efforts to engage Black adults in research and cancer prevention activities who live in low-income communities include poverty [11], limited available transportation [12], distrust of White researchers [13], literacy challenges [14], mobility challenges associated with chronic health conditions most common among Black adults (e.g., obesity and associated joint pain), experiences with racism [15], and limited knowledge of and/or access to technology [16]. Black adults residing in these communities possess lived expertise critical for designing effective, culturally grounded engagement strategies [17]. Yet, Black adults are typically not asked, empowered, nor paid to share this expertise of their lived experience and knowledge with researchers. Furthermore, the diversity and complexity of the above-mentioned SDoHs require more than the existing limited, and often not effective, recruitment strategies to engage Black adults in cancer research, cancer prevention and intervention activities [18].

The purpose of this paper is to describe the University of Florida (UF) Health Cancer Center Community-Partnered Cancer Disparities Research Collaborative (CDRC) model– a human and physical infrastructure model for successfully recruiting and engaging Black adults within low-income communities in cancer prevention, treatment, intervention, and survivorship research and related activities. The CDRC model is (a) informed by the Community-Based Participatory Research (CBPR) Model, (b) designed to be responsive to the earlier-mentioned SDoHs, (c) culturally sensitive, and (d) sustainable and replicable.

## Community-Based Participatory Research (CBPR) Model Informing the UF CDRC Model

The CBPR model informing the CDRC has been set forth in the health promotion and health disparities research literature as a conceptual model for conducting health and health disparities research [19]. This model involves scientific researchers and community stakeholders engaging in systemic and collaborative research as equitable partners who share their expertise and all decision-making for the common purpose of educating, improving practice, and/or bringing about social change [20–23].

The CBPR model requires (a) involving community members as equitable partners in *all* research components, from conceptualization to making meaning of research findings, (b) recognizing that the target community members have expertise that their academic research partners do not have and vice versa, (c) promoting community empowerment through bringing sustainable resources to the community, (d) disseminating research findings to the target research community, including through townhall meetings, (e) including community member research partners on publications from the research for which they were partner researchers in conducting, and (f) paying community member research partners a wage that is equitable and representative of their needed expertise [24–27].

Systematic efforts to evaluate the usefulness of the CBPR model when conducting cancer research in Black communities suggests that this model is useful for such research [28]. For example, a randomized controlled trial to reduce cancer risk among African Americans was conducted that involved community members of African American churches as research partners. Findings from this study regarding the views of these community member research partners about their research participation included high levels of perceived trust in the research project and the project team, benefit from involvement with the project, satisfaction with the project and the team, and low level of perceived burden associated with participation in the trial [29].

While there are several existing studies that discuss the utilization of CBPR principles to facilitate culturally tailored interventions [30–32], few expand explicitly on the human and physical infrastructure used in conducting this research. This infrastructure-driven model extends beyond conventional CBPR applications by institutionalizing a scalable partnership framework through both human and physical community assets.

## Implementation of the UF CDRC Model in Low-Income Communities in East Gainesville, Florida

The CDRC model has been implemented in low-income communities within East Gainesville, Florida over the past 2 ½ years. The implementation of this model has demonstrated that the CDRC model is an effective, replicable, and sustainable model for successfully engaging Black adults living within low-income communities, such as those in East Gainesville, Florida, in quantitative and qualitative cancer research and related activities (e.g., cancer education activities).

A central tenet of the CDRC model is that cancer research and clinical trials are conducted in a culturally sensitive manner [33]. The indicators of cultural sensitivity are (a) conveying cultural competence in ways that enable others different from you to feel comfortable, respected, and trusting in your presence, (b) being aware of and accepting of cultural differences, (c) being aware of and trying to rid yourself of common racism-related biases and stereotypes, and (d) empowering those in relationships with you who have or perceive having less power than you (e.g., empowering patients) by asking, listening to, and acting on their opinion [34].

## East Gainesville—The UF CDRC Model Implementation Site

Black residents make up 20% of the total county population in Gainesville, Florida, which is located in Alachua County, FL [ 35 ]. Black residents are reported to have a lower median income ($30,123) compared to White residents ($54,112) in Gainesville. In Alachua County, the life expectancy of Black men and women are reported to be 71.3 years old and 77.3 years old, respectively, which is notably lower than the average for Alachua County men (75.5 years old) and women (80.7 years old). Concerningly, cancer has been identified as the leading cause of premature death for Black residents in Alachua County [ 35 ].

Data from the NCI-designated UF Health Cancer Center provides cancer-specific statistics for Black residents in East Gainesville. Within East Gainesville, the overall incidence of cancer is reported as 530.9 per 100,000 [36]. This is greater than the Alachua County (473.7 per 100,000) and Florida (471 per 100,000) cancer incidence rates [37]. The mortality rate for cancer in East Gainesville is 233.4 per 100,000 [38]. This is also greater than the Alachua County (152.3 per 100,000) and Florida (133.4 per 100,000) cancer mortality rates [39]. Within East Gainesville, the most prevalent age-adjusted cancer types among Black residents were reported as prostate (157.1 per 100,000), breast (114 per 100,000), colorectal (62.6 per 100,000) and uterine (34.6 per 100,000) [36]. The most prevalent age-adjusted cancer mortality rates among Black residents were reported as prostate (67.2 per 100,000), lung (33.2 per 100,000), breast (28.1 per 100,000) and colorectal (25.3 per 100,000) [38]. These cancer incidence and mortality rates in East Gainesville provide further support for the necessity of a CDRC human and physical infrastructure model that is culturally tailored and embedded within the low-income communities for Black residents.

## Human Infrastructure of the UF CDRC Model

**Fig. 1:**
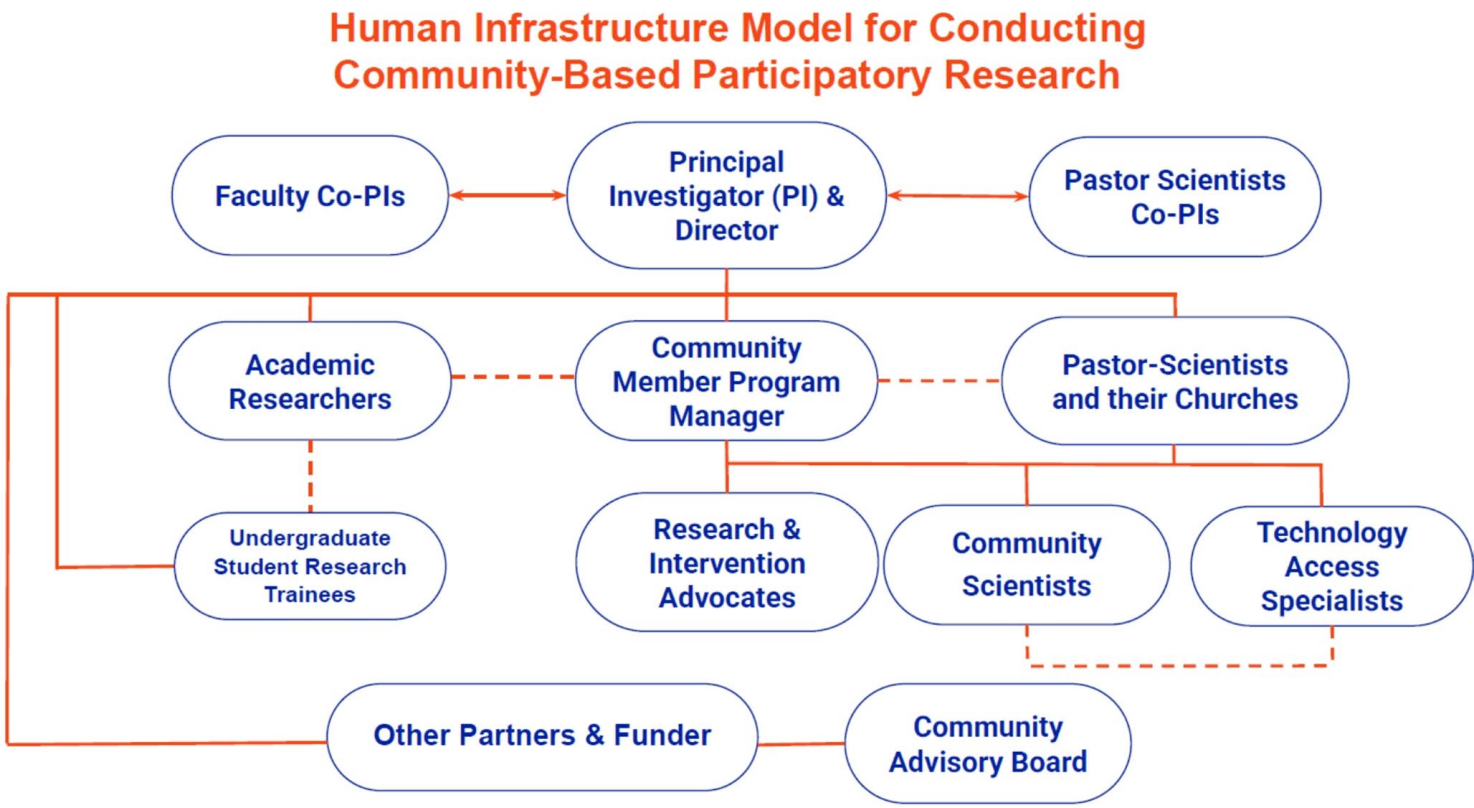
Human infrastructure model for conducting community-based participatory research

The human infrastructure of the CDRC Model as tailored for implementation in Black low-income communities in East Gainesville includes multiple research members and groups and is presented in the CDRC organizational diagram labeled Fig. 1. Below are brief descriptions of these members and groups in the CDRC model.

### Leadership Team

This team consists of a faculty member principal investigator (PI)/Director of the CDRC, five Co-PIs, two pastor Co-PIs called “pastor scientists”, and the manager of the CDRC. These individuals work collaboratively to identify and design research studies, with input and final approval from all other members of the CDRC. This identification and designing of research studies is an iterative process. The faculty members are all members of the UF Health Cancer Center and one of them is the Director of this center’s Biostatistics Core.

The two pastor scientists are each a pastor of a predominantly Black church among the churches in East Gainesville constituting the CDRC. The CDRC manager is a resident of a Black community in East Gainesville and is a retired administrator in the local city government. The PI/Director of the CDRC serves as the conduit of most information shared to and from the faculty Co-PIs and the Pastor Scientist Co-PIs. However, representatives of the faculty Co-PIs as well as the other members of the leadership team meet monthly with the Research Implementation Team.

#### Roles of the PI/Director

The major roles of the PI/Director are to (a) work collaboratively with the Leadership Team to design cancer-focused studies identified by and/or sanctioned by Black adults living in any one of the Black low-income communities in East Gainesville (i.e., the target communities), (b) meet regularly with all of the groups and leaders who constitute the CDRC to ensure that research studies are implemented as designed and in a culturally sensitive manner, and (c) recognize and show appreciation to all CDRC members and groups for the work that they do. The PI/Director also keeps the leadership of the UF Health Cancer Center informed of the research and other activities of the CDRC and forges the building of positive relations between this academic medical center and the target Black communities. For example, the PI/Director and other members of the UF Health Cancer Center have frequently attended church service of one or more of the churches participating in the CDRC.

#### Roles of the Five Faculty Co-Principal Investigators

These faculty co-principal investigators assume major roles in designing and conducting the studies of the CDRC, writing papers on the results of these studies for publication, and presenting the results of these studies at professional conferences and in community settings. One of these investigators is a biostatistician who assumes the leadership in designing data management systems and performing the data analyses for CDRC spearheaded studies.

#### Roles of the Pastor Scientist Co-PIs

These pastor scientist Co-PIs consult on planned research studies, coordinate with the other Pastors Scientists to obtain needed research documents and support letters for grant proposals and provide insights on the results from research studies. They also consult with the PI/Director on strategies for addressing research challenges such as engaging Black men in CDRC research studies. Finally, these Co-PIs co-chair the Community Advisory Board.

#### Roles of the Community Member Manager

The community member manager has several important roles that include: (a) overseeing recruitment and enrollment of research participants from among the target communities, (b) assisting with the training of pastor-selected individuals from the CDRC churches (the churches led by the pastor scientists) to be community scientists—the persons who recruit, enroll, and collect data from participants in CDRC studies, (c) collaborating with the PI/Director weekly on research progress and challenges, and (d) communicating with the PI’s/Director’s Administrative Research (AR) Team and the Pastor Scientists regarding any assistance or resources needed from them to ensure adherence to the timeline for each study spearheaded by the CDRC.

### Research Implementation (RI) Team

The RI team includes (a) 10 Pastor Scientists, including the two who are Co-PIs, and their respective churches (called CDRC churches), (b) a Community Scientist and Technology Access Specialist from each of the 10 CDRC churches, (c) six Cancer Research and Intervention Advocates (CRIAs), all of whom live or work in one of the target communities and identify as a cancer survivor, and (d) the PI’s AR Team, who devote about 30% of their time to the work of the CDRC—work that primarily involves documenting, communicating, coordinating, and overseeing activities that support the work of the RI Team in general and the PI in particular. The RI Team meets monthly, and the chair of these monthly meetings is the Community Member Manager of the CDRC. The purposes of these meetings are to (a) discuss the progress of research studies and cancer prevention interventions and activities, and the related progress of identifying strategies to identify and reduce barriers to recruitment and enrollment, (b) plan cancer education and prevention activities, (c) discuss preliminary research findings, and (d) share information from the director of the UF Health Cancer Center conveyed directly by them or via the CDRC PI/Director regarding ways that the CDRC can best support the mission of the UF Health Cancer Center and vice versa.

#### Roles of the 10 Pastor Scientists

In addition to participating in the monthly RI Team meetings, the Pastor Scientists select and support the Community Scientists and Technologist Access Specialists in implementing their roles at their respective churches. Additionally, the Pastor Scientists announce all activities of the CDRC during their church services and invite members of their church and attendees to their community-based activities to participate in CDRC research and education activities. They also assume leadership roles in semiannual “Power Over Cancer” Gatherings that primarily focus on learning about various cancers, ways to prevent cancers, new cancer treatments, and research opportunities. Notably, these Gatherings are spearheaded by the CDRC in collaboration with the leadership of the UF Health Cancer Center.

#### Roles of the Community Scientists and Technology Access Specialists

The major roles of the Community Scientists are recruiting Black research participants from the target Black communities and then obtaining informed consent and collecting research data from these participants. Their data collection role involves assisting research participants in using iPads to complete research questionnaires and measuring these participants’ weight and height. The Community Scientists also registers people attending each of the “Power Over Cancer” Gatherings and assist attendees in completing quality improvement questionnaires to evaluate the usefulness of these Gatherings. Currently, only one of the Community Scientists identifies as a cancer survivor.

Given that it is more convenient or preferred by some research participants to provide feedback on the CDRC and planned cancer research studies via Zoom rather than via questionnaires, a Technology Access Specialist is positioned at each of the CDRC churches to assist research participants with using Zoom. To determine need for this assistance, the Community Scientist administer a researcher-constructed Zoom Knowledge Questionnaire to all enrolled research participants. The Community Scientist refers those participants who miss items on this test to one of the Technology Access Specialist. The specific roles of the Technology Access Specialist are to teach research participants to use Zoom on their technology devices, assist them with using it as needed, and arrange for them to have access to technology at one of the CDRC churches or at the earlier mentioned non-church multipurpose building that also serves as a CDRC research site. The Community Scientist and Technology Access Specialist meet twice a month with the CDRC Community Member Manager to address their questions (e.g., How to deal with participants who do not show up for or arrive late for their scheduled research enrollment session?) and give them needed information such as updates to Zoom settings and/or changes in research protocols approved by the UF Institutional Review Board.

#### Roles of the Cancer Research and Intervention Advocates (CRIAs*)*

The CRIAs are Black cancer survivors whose important roles in the CDRC include recruiting Black cancer survivors to participate in the research of the CDRC, providing input on research needed to address the concerns of cancer survivors, and co-leading support groups at the “Power Over Cancer” Gatherings—support groups for other cancer survivors and individuals at risk for cancer, groups interested in learning ways to prevent getting cancer, and support groups for family members and friends of cancer survivors. At the “Power Over Cancer” Gatherings, the CRIAs also serve on educational panels with other cancer survivors, oncologists and other cancer care providers, social workers, and psychologists to answer anonymous and openly asked questions from the mostly Black attendees at the Gatherings. The PI/Director of the CDRC and the CDRC manager have bi-monthly meetings with the CRIAs to discuss any support needed to facilitate recruitment of cancer survivors to be research participants and people to be attendees to the Gatherings.

#### Roles of the PI’s Administrative Research (AR)Team

The AR Team includes a Research Manager, Data Manager, and Community Intervention and Research Coordinator. The AR Team Research Manager collects all administrative data and information needed to make sure that all employed CDRC members are paid and are able to respond to questions from any member of the CDRC after consulting with the PI/Director or another appropriate member of the Leadership Team. The Data Manager makes sure all data is protected and prepared for data analyses and trains the Community Scientists to execute their data collection roles. The Community Intervention and Research Coordinator (a) works collaboratively with the PI/Director and the CDRC Manager to make sure that community-based, cancer-focused research and education activities occur as planned, (b) oversees conducting of focus groups to evaluate the CDRC and analyzing of focus group data, (c) makes sure that all community members and others who are involved with CDRC studies undergo training required of them by the IRB, and (d) ensures communication of information among all CDRC members.

### The Health Disparities and Health Promotion Undergraduate Research Interns

The undergraduate research interns are culturally diverse and enrolled in the Health Disparities and Health Promotion Research Internship for undergraduate students at the University of Florida. This internship is taught by the PI/Director of the CDRC. These interns receive course credit for their training in CBPR facilitated research and engagement and they choose aspects of the CDRC in which they want to participate such as assisting with (a) recruiting and enrolling research participants, (b) analyzing focus group data, (c) co-facilitating the training of Community Scientists, and (d) executing various logistical roles at the “Power Over Cancer” Gatherings. They also attend CDRC team meetings of interest to them, conduct literature reviews relevant to the research of the CDRC, and assist with planning CDRC studies.

### CDRC Community Advisory Board (CAB)

The CAB consists of community leaders, is co-chaired by the two Pastor Co-PIs, and meets quarterly. The roles of the CAB are to (a) identify community leaders and organizations to serve as partners of the CDRC, (b) assist the Community Scientists and CRIAs with the recruitment of Black men to be research participants and attendees to the “Power Over Cancer” Gatherings (e.g., the Power Over Prostate Cancer Gathering) and (c) provide feedback on each planned CDRC study and share their views on what results of a CDRC study mean.

### CDRC Partners

One of the partners of the CDRC is the Community Outreach and Engagement division of the UF Health Cancer Center. This division consists of faculty and staff who engage in cancer education and screening activities, and it targets underserved individuals in several counties served by the UF Health Cancer Center. It partners with the CDRC in conducting Power Over Cancer Gatherings and collecting data to evaluate their impact.

The other CDRC partner is the leadership team of the NCI designated UF Health Cancer Center. This team provides funding for the operation of the CDRC and assists with submitting external grant proposals to obtain funding to support the research spearheaded by the CDRC.

## Physical Infrastructure of the CDRC

The physical infrastructure of the CDRC consists of the 10 Black churches pastored by the Pastor Scientists and a multipurpose building owned by UF. These physical infrastructures are in low-income Black communities, all of which are near the UF Health Cancer Center. Each has a large CDRC sign at its entry and private spaces where Community Scientists carry out activities to facilitate participant enrollment and data collection activities. Each private space has a scale and height rod for Community Scientists to collect height and weight measurements. Additionally, several of the CDRC churches have technology spaces where Technology Access Specialists facilitate Zoom education trainings with enrolled participants.

## Operation of the UF CDRC

The Leadership Team and Research Implementation Team of the CDRC meet monthly for the Leadership Team to share research study and grant ideas and related activities (e.g., cancer education activities) informed by the local county’s needs assessment. Input on these ideas and activities are provided by the Research Implementation Team, who suggest research ideas and cancer-related prevention and survivorship ideas to the Leadership Team. Discussions at these monthly meetings ultimately result in specific research projects and activities to prevent cancer and promote cancer survivorship. The Leadership Team writes protocols for these research projects and activities and obtains any required approvals of these protocols from the UF Institutional Review Board. The Research Implementation Team uses these protocols to guide their participant recruitment, enrollment, and data collection activities.

Some sub-group meetings occur outside of the aforementioned meeting. These meetings are: (a) bi-monthly faculty co-PI meetings, (b) weekly meetings of the PI with the Community Program Manager, and the PI’s Administrative Research Team, (c) bi-monthly meetings of the PI/Director and the undergraduate interns in the Health Disparities and Health Promotion Research Internship, (d) monthly meetings of the CRIAs, (e) quarterly meetings of the PI/Director and CAB, (f) quarterly meetings of the PI/Director with the Director of the UF Health Cancer Center, and (g) and annual CDRC Showcase meetings to which all involved with the CDRC, individuals who have been research participants in a study spearheaded by the CDRC, members and leaders of the target and other local communities, and leaders of UF are invited to attend. The purpose of the CDRC Showcase meetings is to share the achievements of the CDRC and its impact on those who participate in it.

Notably, the Community Manager and members of the Research Implementation Team, except the student interns, receive funding from the grant awarded to support the operation of the CDRC. The Pastor Scientists receive a small honorarium for their contributions to the CDRC and their churches receive an honorarium for the added cost of electricity and janitorial service related to hosting data collections and other CDRC activities. All research participants are paid for their research participation at an amount that covers their time and travel expenses.

## Activities of the CDRC

The activities of the CDRC include cancer research and education activities conducted in the target communities in East Gainesville, which is in close proximity to the UF Health Cancer Center. These research activities include:

- recently completing a study involving 403 Black adults who are cancer survivors or at risk for cancer to identify the demographic, health, and behavior correlates of each of these groups and to determine the predictors of each group’s physical and psychological quality of life,
- conducting a virtual focus group study with 50 Black women breast cancer survivors to identify the specific emotional and psychological support behaviors that they need from their partners/spouses, other family members, and health care providers,
- submission of two National Institutes of Health research grants– one focused on testing a health promotion intervention program for preventing colorectal cancer among Black adults in low-income communities, and one focused on the roles of stress, gratitude, and allostatic load in breast cancer survivorship, and.
- recently hosting a CDRC Showcase to highlight the ongoing research of the CDRC and the community members and UF members who work collaboratively to conduct this research, which was attended by all members/groups within the CDRC, residents of East Gainesville and the other areas Gainesville, members of the local social media, leaders of the UF Health Cancer Center and other leaders at UF such as the Dean of the College of Medicine and the UF Provost.
- helping community members navigate to current cancer and health-related studies at the UF seeking to promote health among Black adults.

The cancer education activities include hosting several “*Power Over Cancer Gatherings”* in the target low-income Black communities. These activities are called “gatherings” because during slavery, enslaved Black people were not allowed to gather; and when the last state freed Black people (i.e., Texas), many diverse people gathered in this state to celebrate freedom of all enslaved Black people and others [40, 41]. At the Power Over Cancer Gatherings, African Americans/Black adults have the freedom to gather for the purpose of learning information to help prevent and survive/thrive from the cancers that are most prevalent among them and/or result in them dying at a higher rate than other racial/ethnic groups.

The specific Gatherings that have been held are (a) a Power Over Breast Cancer Gathering, (b) a Power Over Prostate Cancer Gathering, (c) a Power Over Colorectal Cancer Gathering, and (d) a Power Over Ovarian and Cervical Cancer. These Gatherings are 5.5 h long and are held on a Saturday—a day that most members of the target Black communities can attend. These Gatherings are held at one of the Black churches in the CDRC that in addition to its sanctuary, has large break-out rooms and a large meeting room. Approximately 150 to 200 persons have attended each of the past Gatherings, among whom are cancer survivors, individuals at-risk for cancer, adult family members of cancer survivors, cancer researchers and providers, and leader of the UF Health Cancer Center.

Each Power Over Cancer Gathering includes the following activities:

- a brief presentation by each of two oncologists—one who talks about ways to prevent the targeted cancer, and one who talks about the most effective treatments for the targeted cancer,
- public and anonymous questions from the audience at the Gathering to the oncologist presenters,
- break-out group discussion sessions for the audience that are co-led by a cancer survivor and a culturally-sensitive health professional who is knowledgeable about the target cancer, empowering of all in the session to share in community, supportive and engaged in active and compassionate listening,
- a panel session at which panelists consisting of cancer survivors, spouses and family members of cancer survivors, oncologists, other cancer care team members, and a psychologist to answer publicly and privately asked questions from the audience,
- a time for all attendees to visit research tables where cancer and health researchers explain their research and invite these audience members to sign-up to be research participants,
- completion of a quality improvement questionnaire by the audience to assess their views about the helpfulness of the Gathering, desired changes in it, and desired future Gatherings, and.
- a drawing for prizes followed by dissemination of catered healthy to-go food boxes.

The helpfulness ratings on the quality improvement questionnaires completed by the community-member attendees at each Power Over Cancer Gathering ranged from 4.8 to 5.0 on a 1 to 5 rating scale where 1 = *Not at all helpful* and 5 = *Extremely helpful*. Feedback in the invited comments section of these questionnaires included that the gathering was great, the break-out group discussion sessions needed to be longer than 75 min, and there is a need for the Gatherings to continue because they are educational and provide needed support for others and one’s self. The future desired “Power Over Cancer” Gatherings identified on the quality improvement questionnaire were testicular, bone, and uterine cancer; consequently, there is much support for continuing the “Power Over Cancer” Gatherings and having them address these identified cancers.

The activities of the CDRC are intentionally developed and implemented to address key SDoHs impacting members of the target community. See Table 1.

**Table 1:** Summary table of key social determinants of health and CDRC strategies/actions to address each social determinant of health

Social Determinant of Health	CDRC Strategy/Action
Low-income/poverty	Hiring community staff
Health literacy	Power of Cancer gatherings
Technology literacy	Technology trainings
Research mistrust	Community engagement
Health access	Connection to UF Health cancer providers, mobile units

## CDRC Implementation Challenges and Strategies Used to Overcome Them

Three primary challenges were encountered when implementing the CDRC in low-income Black communities within East Gainesville (i.e., the target communities). These challenges were, and ideally should be, effectively addressed in the monthly meetings involving all members of the CDRC or the faculty and community Co-PIs, depending on the issue. Following are descriptions of the three primary challenges encountered and culturally sensitive strategies used to address each challenge.

1. **Trust building with the community members.** As in many cities with predominantly White universities, there is a lack of trust of White researchers and university leaders that is associated with historical abuse of Black adults in research (e.g., the Tuskegee Study) and the common practice of helicopter research (i.e., research aimed at getting needed data in Black communities and leaving no structure or resources in these communities to benefit them (Scharff et al., 2010; Hughes et al., 2017). The strategies used to effectively overcome this challenge included the following: (a) inclusion of Black faculty among the faculty Co-PIs and inclusion of Black pastors whose churches are in the target communities as Co-PIs, (b) receiving continuous annual funding of the CDRC by the Director of the UF Health Cancer Center, (c) hosting all major CDRC activities (e.g., the “Power Over Cancer” Gatherings) in churches within the target communities and consistent participation of the leadership of the UF Health Cancer Center in all of these activities, (d) adherence to the community-based participatory research model, which requires that community members be involved in every aspect of research and related activities implemented by the CDRC, and (e) inclusion of members of the target community in major activities of the UF Health Cancer Center.
2. **Credentialling of community members as Community Scientists**. The term *“Community Scientists*” was created by the PI and Director of the CDRC to facilitate respect for community members trained and credentialed to be research partners. The credentialling process involved (a) engaging in the UF IRB training for researchers and passing the test constructed by this IRB to assure acquisition of the knowledge provided via this training, and (b) undergoing training by the PI and research staff for the purpose of learning how to execute their specific research roles such as obtaining informed consent from study participants. The challenge encountered was in preparing the community scientists to pass the UF IRB-required training. The strategies used to overcome this challenge were: (a) convincing the IRB to allow a PowerPoint of their required training be conducted by the PI and Administrative Research Team, followed by completion of the IRB-required training acquisition test online as is done by any UF personnel, and (b) implementing this training in a culturally sensitive way that included explaining what terms meant, taking as much time as needed to answer trainees’ questions, and conducting the training in a relaxed setting (e.g., a church).
3. **Inspiring consistent attendance of Pastor Scientists to UF CDRC meetings. **The Pastor Scientists and faculty who are part of the CDRC Leadership Team are extremely busy; thus maintaining attendance meetings at required leadership roles was a major challenge. This challenge was overcome using the following strategies: (a) reducing the frequency of meetings the Pastor Scientists are required to attend, (b) holding required meetings via Zoom, (c) corresponding with Pastor Scientists frequently and re-iterating the importance of their contributions to the CDRC, (d) providing opportunities at meetings for Pastor Scientists to share upcoming activities at their churches, (e) including in meetings a discussion of how the research and educational activities of the CDRC will ultimately improve survivorship and prevent cancer in the target communities, and (f) presenting data evidencing the high cancer incidence and mortality rates among Black adults in the target communities.

## Indicators of Successful Implementation of the CDRC

There are several indicators of the successful implementation of the CDRC in Black communities within East Gainesville (i.e., the target communities). Following are brief descriptions of these indicators.

1. **Successful recruitment of study participants in the target communities**. It is notable that 403 Black adults (33% were cancer survivors and 67% were at risk for cancer) living in the target communities were successfully enrolled in the major study of the CDRC within its first year. Particularly notable is that 86% of the cancer survivors and 95% of the participants at risk for cancer had never participated in a research study of any kind. Of the above-mentioned 403 Black adults, 78% identified as cisgender women (22% as cisgender men), the mean age for cisgender women was 63, whereas the mean age for cisgender men was 63. The mean body mass index (BMI) for cisgender women was 34, whereas the BMI for cisgender men was 30. Additionally, based on BMI data, 70% of the cisgender women cancer survivors and 57% of the cisgender men cancer survivors had obesity; 63% of the cisgender women at risk for cancer and 54% of the cisgender men at risk for cancer had obesity. Given that obesity is a risk factor for cancer, it is important that this disease was identified as a major problem in the target community. This ongoing study focuses on (a) identifying the SDoHs and individual level factors that predict getting screened for the common cancers, engaging in healthy lifestyles behaviors, and quality of life among these adults, (b) assessing the levels of these variables, and (c) determining if the predictors and levels of these variables differ for cancers survivors compared to individuals at risk for cancer. In this large ongoing study, individuals at-risk for cancer are those who have a close relative with cancer, have obesity, engage in unhealthy eating and low levels of physical activity, consume one to two drinks of alcohol per day, and/or regularly use tobacco products.
2. **Successful recruitment of Black men study participants. **The low participation of Black men in cancer research has been well documented (Sheppard et al., 2021). In implementing the major study of the CDRC in the target community, 22% of the enrollees in this study are Black men. This is deemed a success, given that less than 7% of participants in clinical trials are Black men (Jaber, 2024).
3. **Consistent high attendance of Black adults from the target community in CDRC education and research activities**. Across the four earlier mentioned “Power Over Cancer” Gatherings, the attendance of Black adults from the target communities remained consistently high. It is also notable that an average of 50% of the attendees at these Gatherings signed up to participate in the research advertised at them by faculty researchers and community scientists.
4. **Scientific productivity. **Within one year of implementing the CDRC, three cancer-focused studies exclusively involving Black adult participants in the target community were launched. Additionally, CDRC PIs were awarded two NIH grants that identified the CDRC as a support structure for recruiting Black participants.
5. **Statewide recognition.** The PI’s CDRC-related efforts were recognized by the Florida Blue Foundation Sapphire Award. This is a very prestigious and annual award recognizing excellence and innovation in community health, leadership, and programs across Florida.
6. **Community recognition**. In addition to statewide recognition, the efforts of the CDRC have been recognized by the community partners. See Table 2 for testimonials from specific community partners.

**Table 2:** Community partner testimonials

Role	Testimonial
Pastor	“The UF Cancer Disparity Research Collaborative (CDRC) is making a remarkable difference by uniting healthcare professionals, churches, and academics institutions to confront cancer disparities head-on. Their commitment to equity and collaboration is transforming how communities understand and respond to health challenges. By bridging the gap between science, spirituality, and service, the CDRC is creating a model of holistic care that uplifts every voice. This impactful work is not only addressing cancer disparities-it is building a healthier, more united future for all.”
Technology Specialist	“From a technical experience, the CDRC established an awesome technical support system for participants and caregivers. Participants were very pleased with the technical assistance received with the use of the IPAD. They were taught how to use ZOOM for the first time and assisted with solving WI-FI issues. It is very rewarding to see the excitement from participants learning how to use an IPad for the first time in CDRC.”
CRIA	“I am a cancer survivor, serving as a Cancer Resource Intervention Advocate (CRIA) with the Cancer Disparities Research Community Collaborative (CDRC). This has been both personally meaningful and professionally fulfilling. The role of CRIA has allowed me to turn the lessons of my personal journey into purposeful, compassionate, and community-centered support. The work of the CDRC enables me to contribute in a meaningful way to a program that addresses health disparities and systemic inequities in the Black community as it relates to cancer care and outcomes. It also helps ensure that every voice is heard and every life matters.”
Student Intern	“As a student intern and aspiring physician, my experiences working with the CDRC made me more confident in my abilities as a culturally sensitive individual, something I wasn’t able to solely learn from textbooks and school. Hearing tough conversations in focus groups and support sessions allowed me to be comfortable feeling uncomfortable, a trait that I now realize is essential as an aspiring physician. The CDRC showed me that with all that is going on in the world right now, it is more important than ever to find a community and a space where you can make a real difference.”
Community Member	“Made me more aware of getting proper care and getting adequate amounts of sleep/rest. It also made me more aware to become more physically active; stressing less, fearing less; and keeping my doctors appointments.”
Community Member	“The UF Cancer Disparity Research Collaborative (CDRC) has helped me with gaining knowledge about Breast Cancer and other cancers. Additionally, I learned how to cope with my relationship with my partner and gained insight about intimacy. This program has given me the courage to communicate with my doctors and others as a cancer survivor.”

## Demonstrated sustainability of implementing the CDRC

This sustainability is demonstrated by (a) continued active involvement of all of the ten CDRC churches and the community located multi-purpose building that constitute the physical infrastructure of the CDRC model as research sites in the implementation of the CDRC using this model, (b) from year one to year two of implementing the CDRC, the number of church research sites increased from eight churches to ten churches, (c) the continued engagement of student interns with the implementation of the CDRC one to three years beyond their initial one year internship requirement, (d) awareness and use of the acronym “CDRC” by most of the members of the target communities and members of the UF Health Cancer Center, (e) provision of consistent funding of the CDRC implementation by this Center, and (f) ownership of the CDRC by the Pastor Scientists as indicated by shared decision-making, honoring of their community scientists during church services (e.g., praising them and presenting them with monetary gifts to show appreciation for their valuable contributions to implementation of the CDRC), and their provision of supplemental funding of CDRC activities (e.g., payment for food at the CDRC Power Over Cancer Gatherings).

## Future Directions Regarding the Human and Physical Infrastructure of the UF CDRC

The future directions for implementation of the human and physical infrastructure of the CDRC include finding a long-term funding source. It is expected that this funding (i.e., approximately $200,000 per year) will be found given (a) the training opportunities that the CDRC provides for UF undergraduate and graduate students, (b) the part-time jobs that it provides for community members (e.g., Community Scientists), and (c) the need for Black adults living in low-income communities to participate in cancer research and health research being conducted by researchers at UF. Additionally, junior faculty will be invited to participate in the CDRC and receive mentoring in the conduct of community-based participatory research. The future direction regarding use of the of the CDRC Model includes (a) testing this model in rural Southern communities given the higher cancer rates in these communities than in urban Southern communities [42], (b) publicizing this model via social media as well as refereed journal articles that include community members as co-authors of these articles, and (c) training community scientists and student interns to implement the CDRC and/or serve as CDRC implementation consultants.

## Conclusions

The CDRC model (i.e., the UF Health Cancer Center Community-Partnered Cancer Disparities Research Collaborative model) is a novel replicable infrastructure model for the sustained recruitment and engagement of Black adults within low-income communities in cancer prevention, treatment and survivorship research. The need for this model is evidenced by the underrepresentation of Black adults in general and Black adults in low-income communities in particular in cancer clinical trials and cancer research. The outcomes of such underrepresentation include the development of cancer treatments and prevention and survivorship strategies that are not effective for and/or not engaged in by many Black adults, regardless of their income status.

Most of the work to implement the CDRC using the CDRC model involves and targets Black adults in low-income communities within East Gainesville, Florida, which is in close proximity of the UF Health Cancer Center. Furthermore, the CDRC model is anchored in the CBPR model, which requires that members of a target community are involved in every aspect of the research process and have equitable power to that of their academic research partners.

Notably, the CDRC model has facilitated the (a) enrollment of 403 Black adults from low-income communities who have cancer or are at-risk high risk for cancer and never before participated in any research (b) sustained engagement of Black community members in an ongoing “Power Over Cancer” Gatherings Series at which these community members have enrolled in various cancer research studies, (c) sustained presence of the human and physical infrastructure the target low-income Black communities, and (d) an expanding cohort of faculty, students, and community member researchers who have been successfully trained to conduct culturally sensitive cancer and cancer-related health research and community-engaged research. Thus, the CDRC model is both culturally sensitive and responsive to the national calls for community empowerment models and approaches in efforts to reduce and eliminate health disparities and promote health equity. The CDRC model holds much potential for conducting research that aims to eliminate cancer disparities that disproportionately affect low-income Black communities.
